# Web-based GIS for spatial pattern detection: application to malaria incidence in Vietnam

**DOI:** 10.1186/s40064-016-2518-5

**Published:** 2016-07-08

**Authors:** Thanh Quang Bui, Hai Minh Pham

**Affiliations:** VNU University of Science, 334 Nguyen Trai, Hanoi, Vietnam; Vietnam Institute of Geodesy and Cartography, 479 Hoang Quoc Viet, Hanoi, Vietnam

**Keywords:** Web-based, Health pattern detection, Malaria

## Abstract

**Introduction:**

There is a great concern on how to build up an interoperable health information system of public health and health information technology within the development of public information and health surveillance programme. Technically, some major issues remain regarding to health data visualization, spatial processing of health data, health information dissemination, data sharing and the access of local communities to health information. In combination with GIS, we propose a technical framework for web-based health data visualization and spatial analysis.

**Methods:**

Data was collected from open map-servers and geocoded by open data kit package and data geocoding tools. The Web-based system is designed based on Open-source frameworks and libraries. The system provides Web-based analyst tool for pattern detection through three spatial tests: Nearest neighbour, K function, and Spatial Autocorrelation.

**Results:**

The result is a web-based GIS, through which end users can detect disease patterns via selecting area, spatial test parameters and contribute to managers and decision makers. The end users can be health practitioners, educators, local communities, health sector authorities and decision makers. This web-based system allows for the improvement of health related services to public sector users as well as citizens in a secure manner.

**Conclusions:**

The combination of spatial statistics and web-based GIS can be a solution that helps empower health practitioners in direct and specific intersectional actions, thus provide for better analysis, control and decision-making.

## Background

There is a great concern on how to build up an interoperable health information system of public health and health information technology within the development of public information and health surveillance programme. Technically, some major issues still remain in health sector that relate to health data visualization, spatial processing of health data, health information dissemination, data sharing and the access of local communities to health information (Boulos 2011; Highfield et al. 2011). Pfeiffer and Robinson (2008). Knowing of pattern of distribution enables health practitioners to understand underneath mechanism of diseases development over time and driving factors of those changes. Therefore, the identification and measurement of patterns is an important step in analysing geographic information (Graham et al. 2004; Elliott 2001; Pfeiffer and Robinson 2008).

In comprehensive analysis, a known pattern of disease distribution might reveal the processes underlying the spatial distributions of disease cases, through further analysing physical, environmental and social conditions of the study areas (Kienberger and Hagenlocher 2014; Odongo-Aginya et al. 2005). Spatial patterns can be classified as regular, random, or clustered. Meanwhile, analysis methods are grouped into ‘Non-specific or clustering analysis’ and ‘specific or cluster detection analysis’ (Elliott 2001; Wakefield and Kelsall 2000). McLafferty (2015); Pfeiffer and Robinson (2008) summarized spatial data analysis in epidemiology in their works. Gruebner et al. (2015) used spatial statistics methods to study geographic variability and to disclose patterns of psychological vulnerability and resilience factors in the aftermath of disasters. Goovaerts and Jacquez (2005) detected temporal changes of cancer rates using spatial statistics analysis. (Lin and Zhang 2004); Lin (2004) developed method for spatial clustering test of rare diseases.

**Fig. 1:**
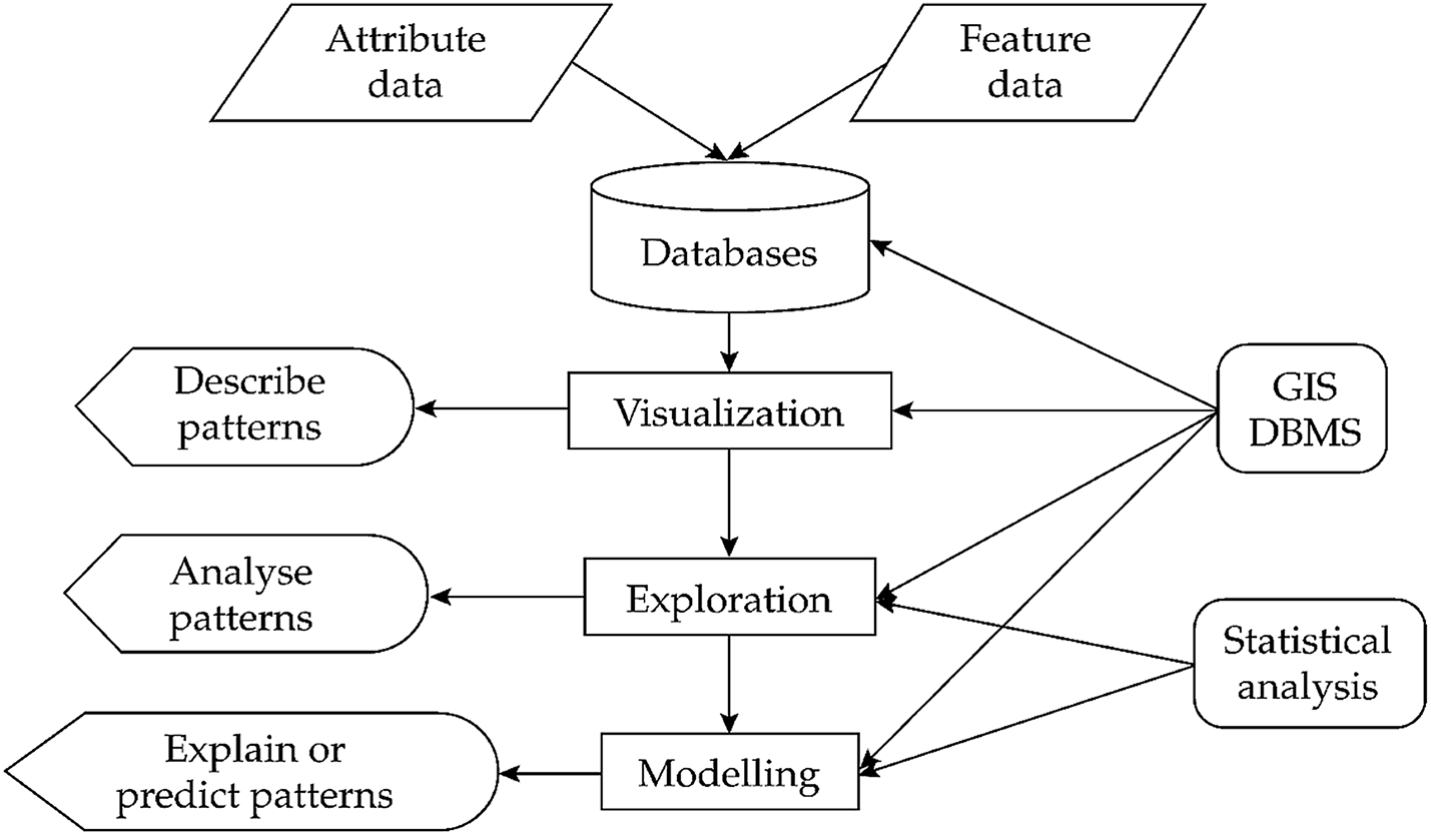
Conceptual of epidemiology data analysis Adapted from (Pfeiffer and Robinson 2008)

Mostly, in epidemiological practices, health data is processed using spatial analysis methods (Auchincloss et al. 2012; Graham et al. 2004). In most cases, geo-referenced data is used, in combination with attribute data that describes the characteristics of disease locations. Based on that, visualization, exploration and modelling can be carried out to assist in decision making (Fig. 1). Despite acknowledging the importance of spatially explicit processes in determining disease risk, the use of spatial information beyond recording spatial location and mapping disease risk is rare (Jacquez 2000). However, the use of spatial information becomes popular with the integration of GIS and statistics package in processing health data (Delmelle et al. 2010) and in opening up data linked to health services (Shoultz 2015). In fact, technical aspect is not an obstacle for the widespread uses of GIS-based health applications, but non-technical aspects remain a major bottleneck (Carroll et al. 2014) that requires a comprehensive strategy for spatially enabled health services.

### Web-based information dissemination

The fast development of Internet and Web-based services helps health practitioners and communities to effectively and efficiently share health information and disseminate risks of diseases (Highfield et al. 2011), even though the services were limited in simple display and query tasks (Longley 2005). Recently, burgeoning efforts have been made to develop more active and dynamic systems and to make Web-based GIS more interactive for end users, such as statistical packages in health data processing and health information dissemination. Technically, geo-technology has enabled the health data sharing and analysis, but socially, it results in a major challenge in protecting patient geo-privacy while allowing analyses of spatial data (Hampton and Fitch 2010). The larger number of data sets become public, the more health information is exposed to communities. Recently, the opening up of health data leads to the development of filter techniques to mask out locations of patient’s location information to preserve confidence. Although enormous efforts have been made, the instilment of sensitivity to geo-privacy and disclosure risk have been unsuccessful (Kounadi and Leitner 2014), particularly in the era of internet.

There is significant demand to exploit current geospatial information infrastructures in health applications (Granell et al. 2014). Researches have focused on the area of Web-based health information and more researchers (academic and non-academic) are using the web for data sharing, research and planning purposes (Lee 2010). Gao (2009) also emphasized the importance of comprehensive representation of heath information to users. Highfield et al. (2011) worked on interactive web-based mapping that provides health data easily accessible for a wide variety of research audiences. The authors urged that the system need to be as interactive as possible, allowing users to dynamically define their research parameters rather than being forced to accept pre-set queries. Boulos (2011) pointed out the application of open map services as Google, Openstreetmap to collect data from communities (crowd-sourcing) and discussed the benefits to get local communities involved in health surveillance. Delmelle and Zhu (2014) recently mentioned the state-of-the art web-based applications in epidemiology and produced an analytical module to map the outbreaks of dengue fever in Colombia, by using accelerated Kernel Density Estimation. Almost all mentioned researches assessed the available Web-based package for health information dissemination, but there are restrictions on spatial analytical capabilities (Delmelle and Zhu 2014) and lack of web-based pattern mapping tool, fostering multi-level participation to support identification of diseases clustering. Even though many web-based health applications dynamically generate view-only maps or interactive maps (Gao 2009) and allow users to study spatio-temporal distributions and evolution of patients (Hammond and Barzyk 2008). However, web-based health pattern detection tools were limited to some specialized researches, using specialized tools in particular a Web-based system (Mills 2008).

This paper focuses on creating web-based spatial processing tool that can be used to measure spatial patterns of the incidence over health data in Vietnam (in this case malaria samples were used). We develop three tools, namely nearest neighbour analysis, K-function, spatial autocorrelation to test the significances of clustering of point distribution. All points are treated in the distribution as if they are all the same they do not distinguish points by their attributes in the first two tools. The third uses aggregated data and takes into consideration both location and locational characteristics. These pattern detection tools can provide preliminary overview on how diseases clustering magnitude be found and will be useful for health practitioners.

## Methods

### Malaria prevalence and pattern

The occurrence of malaria is closely related to environmental, climatic (Odongo-Aginya et al. 2005) and socio-economic conditions (Kienberger and Hagenlocher 2014). Malaria is still a major health problem in the world and Vietnam in particular. Though its prevalence has been reduced, there is great risk of diseases re-emergence to susceptible communities which live in the remote regions, especially those living in border areas between Vietnam and Laos and Cambodia (Hoang 2014) and in highland regions of Vietnam. Most of researches on malaria in Vietnam have focused on malaria epidemiology and achieved many results in prevention of malaria. But the disease is not adequately addressed due to lack of research on disease management, monitoring, detection and early treatment of malaria patients among local communities. A mechanism to share health information throughout communities is an important route to reduce national-wide public health issues (Hoang 2014). This research used malaria distribution as example data for spatial pattern detection tools.

### Web-based spatial analysis tool

Spatial-analytical tools have increasingly been used with epidemiologic data to address the complex spatial nature of exposure-disease relationships. Management of the data is performed using geographic information systems (GIS) and database management systems, and is of relevance throughout the various phases of spatial data analysis. Many applications and GIS platforms demonstrate the application of spatial analysis in epidemiologic research, introducing analytic approaches which integrate GIS and standard statistical software tools to enhance epidemiologic assessments. They provide tools in visualization, exploration and modelling (Abdullahi et al. 2010; Dominkovics et al. 2011; Gao 2009). They cover approaches for identifying spatial clustering of disease, spatial interpolation and imputation methods, geographically weighted and land use regression, and basic spatial statistics useful for studying the spatial distribution of exposure and disease. Even though many Web-based health applications dynamically generate view-only maps or interactive maps, background processing and metadata are often missing (Gao et al. 2009).

In the literature review, several projects have focused on the use of representational state transfer (REST) architectural style and have proved it as an effective and efficient style in Web applications (Arakawa and Kido 2010; Dominkovics et al. 2011; Flemons and Guralnick 2007; Highfield et al. 2011; Kvilekval 2010). In general, clients interact directly with the exposed resources through a uniform interface, materialized basically through the combination of HTTP (hypertext transfer protocol), URI (uniform resource identifier) and standard formats such as HTML (hypertext markup language) and XML (extensible markup language).

### System design

Web-based system provides fundamental tools for visualization, query and for spatial analysis in some cases. Users can interactively define study area and test whether observed distribution and known pattern are statistically different. It also provides an Information communication page. The communication component enables sharing of local/scientific knowledge and guidance of decision makers. The information system will be getting more and more enriched with analysed data by scientists, information verification and feedbacks from the users.

**Fig. 2:**
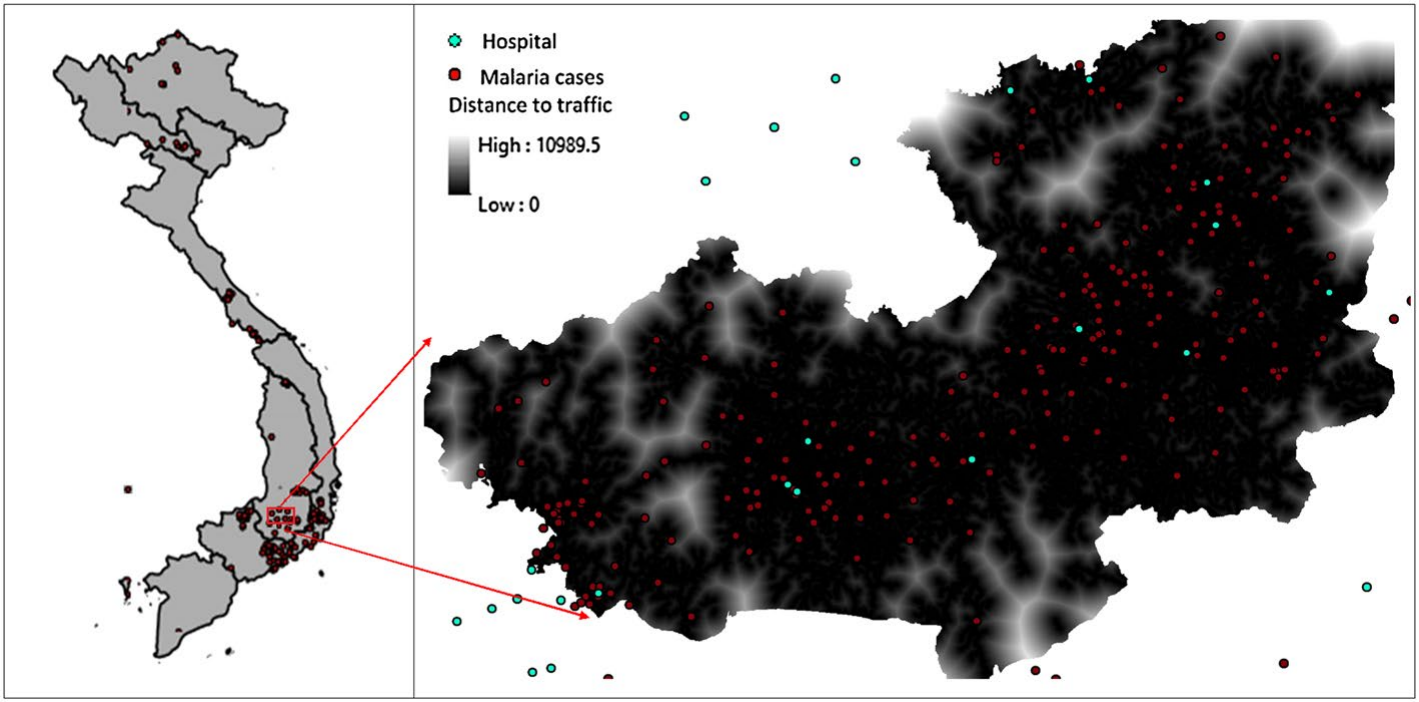
Locations of reported malaria cases

Figure 2 shows conceptual epidemiology data analysis capabilities in GIS, in which the authors propose an open-source framework to deal with pattern visualization and pattern analysis. The criteria for selection of systems embrace many aspects, such as Free and Open source software (FOSS), leverage open standards, hierarchical layering, spatial functionality, query layout, documentation and loading time. Among the two most popular open-source mapping servers UMN Mapserver and Geoserver, Geoserver was selected because of its extension that serves Web Processing Service. From the client side, there are many available frameworks that are sophisticated enough to compare with each other. This research employed GXP library, Openlayers, Extjs and GeoExt. It offers n-tier architecture consisting of: data layer, visualization and processing layer, and presentation layers.

## Case study and data collection

**Fig. 3:**
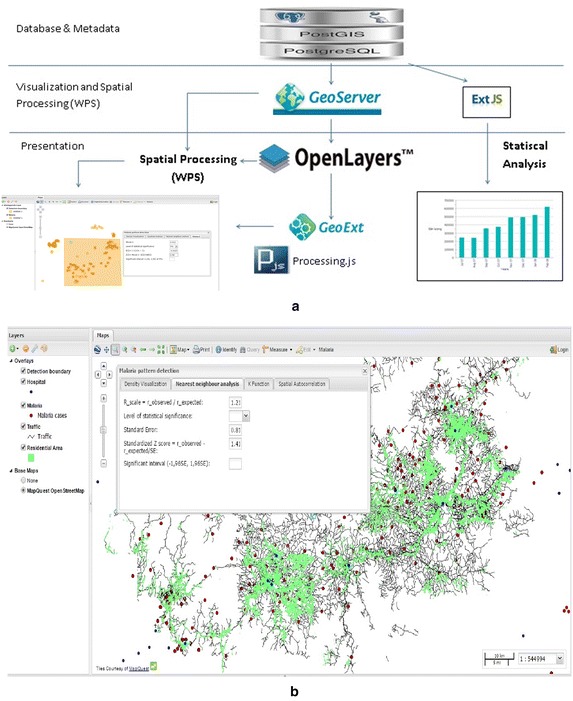
Architectural framework **a** Architectural framework **b** WebGIS interface

A case study was carried out in Lam Dong, Vietnam, a mountainous area in the central highland regions of Vietnam (Fig. 3). The study area is characterized by complex spatial variations in topography and climatic conditions. It is projected as one of the most vulnerable areas to malaria due to more complex topography, lower development index, difficult access to social services, and increasing pressure of population growth and its demand for production land. Furthermore, the combination of livelihoods of local communities (remote farming practices) and lack of mosquito control results in a significant increase in infected cases.

*Data from the web* For the case study, location of malaria cases were collected through internet-based open data, field data collection by open data kit (ODK) field survey and coordinate geocoding from statistical data. In spatial epidemiology, the most common form of data aggregation is sums of diseases cases in each area (Pfeiffer and Robinson 2008). For aggregated data, location is usually a generalisation of study area as centroids of administrative borders. The open malaria data from the last several years for all provinces of Vietnam was downloaded from (http://www.map.ox.ac.uk/explorer/http://www.map.ox.ac.uk/explorer/). Data were filtered to remove unfit cases for the study.

*Data geocoding* Spatial location in maps can be defined by Geocoding tool that enriches a description of a location, most typically a postal address or place name, or matching column value of statistic table to attribute table of spatial data (shapefile). Geocoding provides position of points with a pair coordinate that facilitates spatial analysis in geographic information systems. This method is commonly used throughout health-related research, because health data often only contain low accurate position descriptions like district names and administrative units. (Dominkovics et al. 2011). Malaria cases in 2014 and first quarter of 2015 were collected mainly in the Lam Dong Department of Health and from the National Institute of Malariology Parasitology and Entomology (Fig. 3). Data collected should be taken into consideration with the extent of geographic areas in the study. Analysts often need to determine to what extent the areas surrounding the geographic objects of interest are to be included in their analysis.

*Field data collection by ODK* Additional malaria cases were added to the database using ODK. ODK is a free and open-source set of tools which help to provide mobile data collection solutions with GPS locations and images. ODK is being used to create decision support for technician to define diseases locations and for building multimedia-rich nature mapping tools. Using the ODK development interface, this research was able to develop a core template for the data collection survey (on mobile devices) that was then modified based on the specific data collection needs. Collected data was sent to Tomcat server, backed with PostgreSQL database. With updated points in the database, users can refresh browser for displaying map. Taking advantage of the integrated nature of the ODK Collect survey tool and ODK Aggregate server, surveys could quickly be modified, tested, and released for further testing or data collection.

## Results and discussions

### Density visualization

**Fig. 4:**
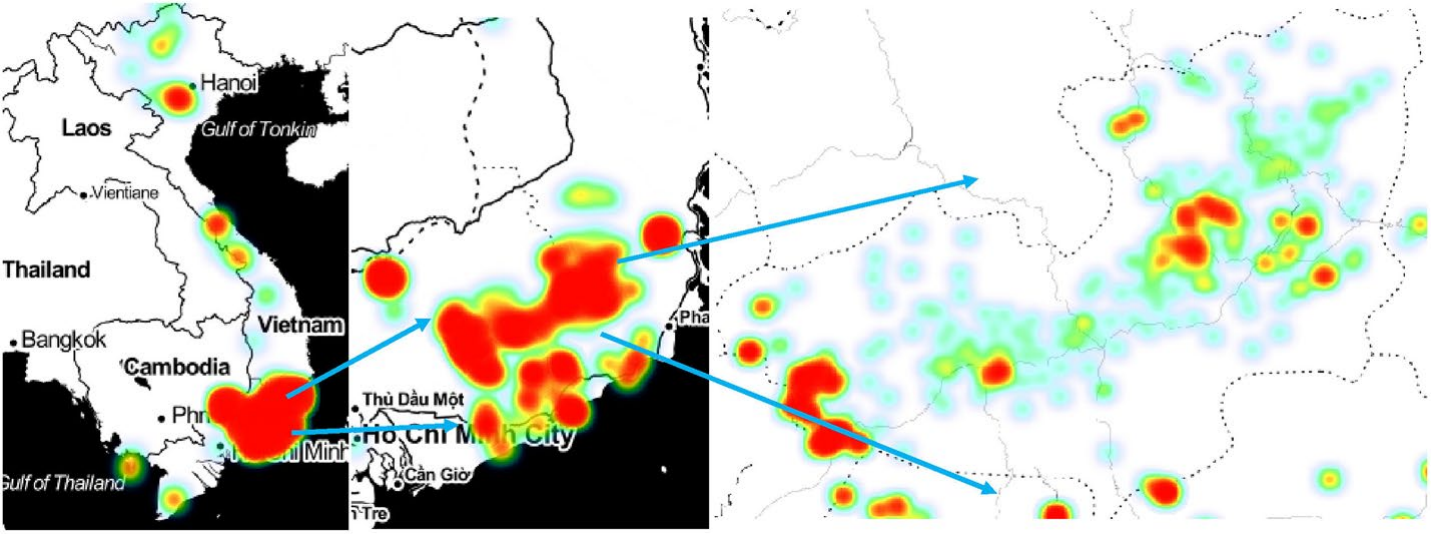
Web-based density mapping

Based on test data, this tool performs a density measurement and visualization that provides a preliminary density visualization of diseases. Results depend on zoom level to show different magnitude of clustering. The processing service performs a heat map web processing service (WPS) service to compute a spatial density map to detect high concentration area of malaria cases. Malaria reported cases were stored in PostGIS and served by Geoserver. When the user makes a change to zoom level, request is sent back to server to recalculate the spatial map with changes in values according to the zoom level (Fig. 4).

### Nearest neighbour analysis

**Fig. 5:**
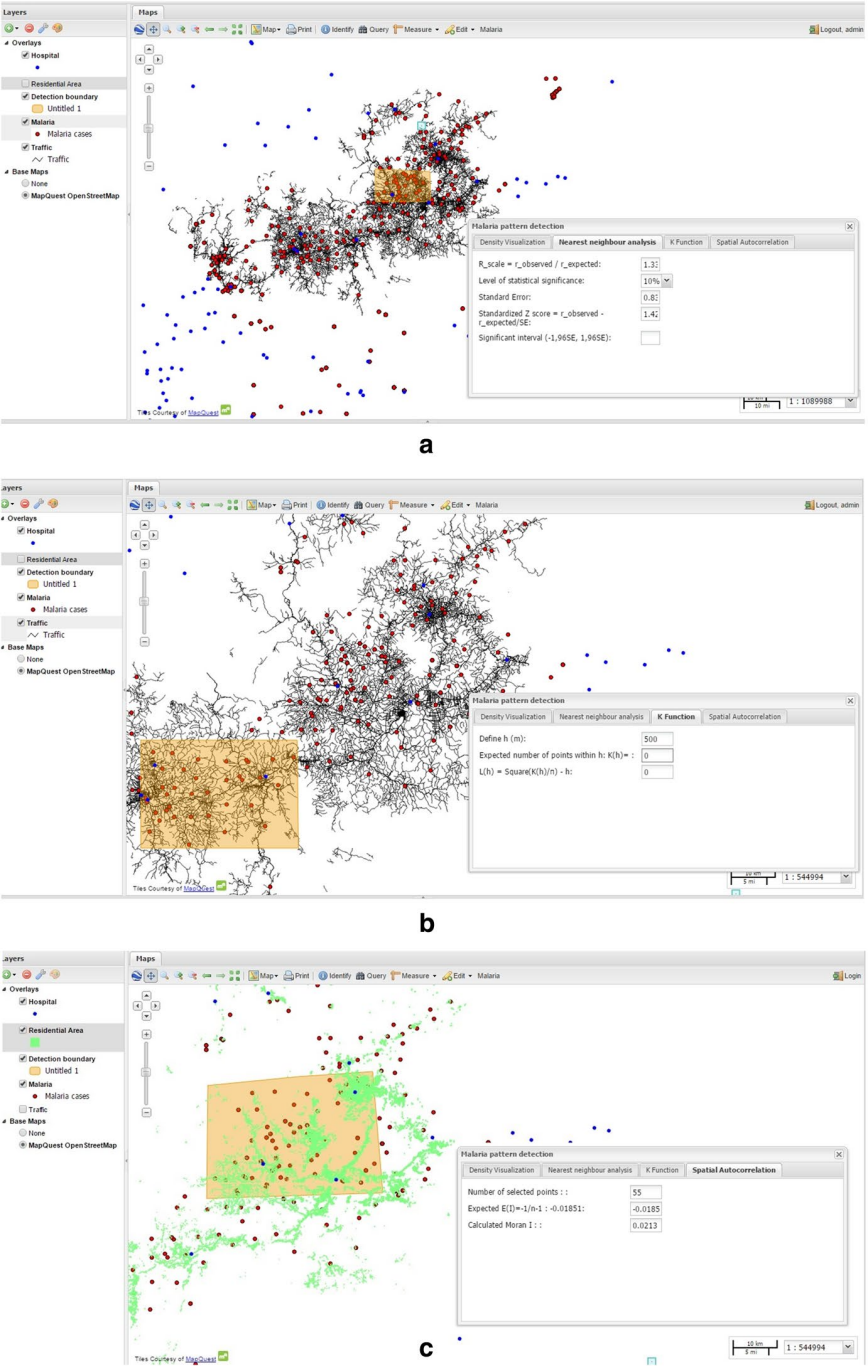
Spatial tests **a** Nearest neighbour analysis **b** K-function **c** Spatial autocorrelation Moran's I

The test is based on comparing the observed average distances between nearest neighbouring points and those of a known pattern (Wong 2005) and is used to estimate the spatial proximity among events. User can draw a box around cluster of points to define boundary of study area, then select level of significance. The tool will calculate R scale, standard error, Z score through calling PostGIS function and compare it to significant interval to check whether the observed distribution is significant different from random pattern. The example showed that Z score is between (−1.96SE– >1.96SE) at 5 % significant level that means observed and random pattern are not statistically different (Fig. 5).

A function was built in PostGIS to calculate sum of nearest distances from selected points that are spatially within area of interest. The final sum of nearest distance is used for R test to check whether the observation is significantly different from random distribution.

- St_contain (geomA, geomB): This function selects all geomA (malaria points) that spatially are inside the bounding box of geomB (box that user draw).
- St_sum, St_Distance: The functions are used to calculate the shortest distances between malaria points and sum the nearest distances up.

With all measured parameters, this tool will calculate D statistic value and D_α_ for K–S test, by calling PostGIS function. The example showed that D statistics is greater than D_α_ = 5 % that means two distributions are significantly different in a statistical sense (Fig. 5a). As in the figure, we defined an area in Lam Dong province and the result was considered to be a non-random pattern at the α = 0.05 level.

### K-function

One of the limitations of the nearest neighbour distance method is that it uses only the nearest distance, as such it only considers only the shortest scales of variation. The K-function provides an estimate of spatial clustering over a wider range of scales. This higher-order analysis based on all the distances between events in the study area and assumes isotropy over the region. The tool starts with drawing a box as a bounding box for malaria point selection, and continues selecting a distance or spatial lag. For each point in the selected area, a buffer with radius equal to spatial lag is drawn and sum of numbers of points within each buffer is calculated.

We build a web-based tool where users can interactively set an area of interest and set value for initial distance (or spatial lag) *h*. We use functions in PostGIS to create buffer for each point and calculate the sum of numbers of point that are spatially within the buffers. Result are stored in an array for graph display and spatial homogenous test. The final sum of the nearest distance is used for R test to check whether the observation is significantly different from random distribution. K(*h*) is then plotted against different values of *h* that describe paired comparison between observations and random patterns.

- St_buffer (geom, spatial lag): Thus function creates buffer for each point with radius defined by a spatial lag.
- St_contain (geomA, geomB): This function selects all geomA (malaria points) that spatially are inside the bounding box of geomB (box that user draw).
- St_sum, St_Distance: The functions are used to calculate the shortest distances between malaria points and sum the nearest distances up.

### Spatial autocorrelation

Neither the nearest neighbour, nor the K-function take point’s attributes into consideration. Different geographic locations rarely have identical characteristics. Because, this is aggregated data, in which one location might contain information on malaria incidences of an area (in this case, areas can be at commune or district level). The number of cases in an area can be used as weight of points in spatial analysis. Spatial autocorrelation is used in considering the effects of both distances between points and their attributes. With spatial autocorrelation, Moran’s I index is used to measure the proximity of location and similarity of the locations. In this study we develop a PostGIS tool to measure Moran’s I, in which data can be either continuous or count data.

Moran’s I coefficient of autocorrelation quantifies the similarity of an outcome variable among points that are defined as spatially related. It measures the proximity of disease points and the similarity of the characteristics of those points (Wong 2005). The tool starts with drawing study area and then by selecting level of significance. It then calculates E(I), Z(I) value through spatially processing distances between points, through calling PostGIS function. This example showed that Z(I) lied between significant interval, so that the observed distribution is not statistically different from random pattern as described in Eq. (1).

1 $$Moran's I = \frac{{ - \mathop \sum \nolimits_{i = 1}^{n} \mathop \sum \nolimits_{j = 1}^{n} w_{ij} (x_{i} - \overline{x} )(x_{j} - \overline{x} )}}{{\text{s}^{2} \mathop \sum \nolimits_{i = 1}^{n} \mathop \sum \nolimits_{j = 1}^{n} w_{ij} }}$$

In which s^2^$= \frac{{\mathop \sum \nolimits_{i = 1}^{n} (x_{i} - \overline{x} )^{2} }}{n}$, n = number of points.

- St_contain (geomA, geomB): This function selects all geomA (malaria points) that spatially are inside the bounding box of geomB (box that user draw).
- St_Distance: The functions are used to calculate the distances between malaria points and weight between point i and j is calculated as w_ij_ = 1/St_distance (geom_i_, geom_j_) w_ii_ is equal to 0.
- St_sum: Sum up attribute values of all points.

Expected E(I) is measured as E(I) = −1/(number of points −1). Cluster pattern, Random pattern and dispersed patterns are detected when calculated Moran’s I value is higher than E(I), equal to E(i) or small than E(I) respectively. In this example, with the calculated score was higher than expected E(I) of −0.185, the malaria points showed a slightly statistically significant departure from a random pattern. This was demonstrated by high score of 0.0213 [a bit higher than random E(I)].

The selection of proper geographic scale for clustering analysis affects the analysis results. Objects may be detected as clustered, dispersed or random depending on the actual scale (in this case zoom scale) we define before proceeding the analysis. In fact, data was geocoded using reported addresses. However, there were missing in documenting correct addresses, then they were aggregated into communes or even into districts. The effect is known as Modifiable aerial unit problem that can radically affect the analysis results.

Indeed, the examples describe three spatial detection tools that compare the observed distribution to random pattern of points, or more specifically pattern of diseases. These functionalities are powerful testing tool to convey message to and to support decision making. By zooming in and out, in combination with clustering tool, users define the boundary of study area and understand changes of analytical results that vary with zoom scale. Nearest neighbour can be used for the global test of clustering, it measures distances between points and compare these distances to known pattern. It has been extended (in comparison to Quadrat statistics) to accommodate second, third, and higher order neighbourhood definitions. The outcome results of this analysis vary depending on spatial scale, because some look clustered in small scale but seem dispersed at large scale. If boundary box size is too small, each polygon may contain a couple of points. On the other hand, if it is too large, each polygon contains many points. This measure does not take into account that different points may be different in how points are represented (due to their differences in characteristics).

The second analysis takes into consideration distances among points and results in plot, describing areas where patterns are clustered or dispersed. All spatial processing of three analyses are done in PostGIS and measured statistics are sent back to client. The third one measures the proximity of location in considering effects of locational characteristics. However, this test considers the population at risk is evenly distributed within the study area and correlation in all directions are to be the same (Moran 1950). This is a drawback of the Moran’s I test. Through the Web based pattern detection tools, end users can interactively define (draw) area for measurement. The tools are three straightforward measurement of clustering and diversion that provide preliminary assessment to disease distribution. More spatial tests should be further incorporated.

## User evaluation

For test and evaluation, in order to improve the quality of the system, and to make the products more useful and usable, we have targeted group of epidemiologist and health practitioners in the National Institute of Malariology Parasitology and Entomology and the National Institute of Occupational and Environmental Health. The prototype was introduced and participants were guided to use the system to detect clustering in Lam dong province as demonstrated in the sections and feedbacks were reported. We collected 19 responses from the participants from both agencies. The questions focused on (1) whether the objectives of the application are relevant (2) user interface (3) are clustering methods useful (4) is the tool easy to convey a message to decision makers (5) what scale selection should be fit for clustering analysis. Values were given in range from 1 as worst to 10 as best. Overall assessment was shown in (Table 1).

**Table 1:**
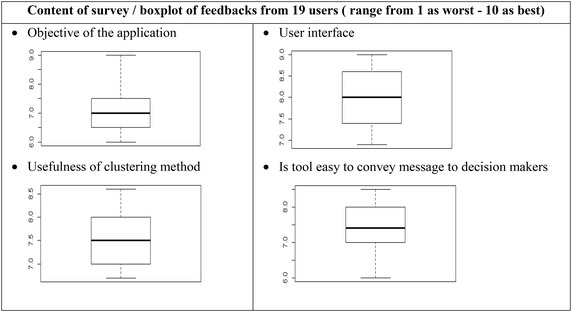
User evaluation

The results from questionnaire indicated the overall satisfaction of the surveyed group in the two organizations. System approach, user’s interfaces, clustering usefulness and easiness to convey message, received upper moderate acceptance from participants, whereas the selection of scale for analysis was discussed among users. The survey revealed that an online clustering application had not been deployed in both agencies and there was relevant demand to have a system that support simple and fast analysis either on the field or indoor. On the other hand, the effects of scale on analysis are significant to conclude whether patterns are clustered/dispersed or randomly distributed. Based on clustering analysis, further studies on variety of driving forces of those pattern will be conducted. The users acknowledge the fundamentality of clustering in epidemiology and discussed on the need to have an interactive tool do the analysis. They commented on integrating administrative boundaries instead of interactively defining the boundary box. The survey results provided useful feedbacks from local administrations who are key people in decision making and local communities who are directly affected by disasters, and can be used for future research and improvement of the system.

## Conclusions

In this paper, the WebGIS system was developed as a tool in promoting a user-friendly approach in detecting malaria pattern in Vietnam. Three pattern detection tools were developed as nearest neighbour, K-function, spatial autocorrelation to visually test health data distribution to known distribution or random distribution. Although, all points are treated in the distribution as if they are all the same they do not distinguish points by their attributes in the first two tools, the results might provide a visualized view on how health patterns are and where incidents are clustered. This system helps health professionals in analysis and decision-making tasks. The success of such pattern measurements mainly depends on the precise collection of malaria point samples and interactive identification of analysis boundary. This system is typically directed toward the widest possible end users (decision makers, managers, local communities) whose contributions may vary considerably. The combination of these benefits may result in direct, specific intersectional actions, and thus provide for better analysis, control and decision-making.
